# Treatment of diabetic nephropathy with *Tripterygium wilfordii* Hook F extract: a prospective, randomized, controlled clinical trial

**DOI:** 10.1186/1479-5876-11-134

**Published:** 2013-05-31

**Authors:** Yongchun Ge, Honglang Xie, Shijun Li, Bo Jin, Jinhua Hou, Haitao Zhang, Mingjun Shi, Zhihong Liu

**Affiliations:** 1 Reasch Institute of nephrology, Jinling Hospital, Nanjing University school of medicine, Nanjing, China

**Keywords:** Type 2 diabetes mellitus, Diabetic nephropathy, Proteinuria, *Tripterygium wilfordii* Hook F (TwHF), Angiotensin II receptor blocker (ARB)

## Abstract

**Background:**

Diabetic nephropathy (DN) is the most common cause of end-stage renal failure. Although angiotensin II receptor blockers (ARBs) can be used to attenuate proteinuria in DN patients, their efficacy remains limited. This clinical trial aimed to evaluate the efficacy of *Tripterygium wilfordii* Hook F (TwHF) extract in the treatment of type 2 diabetes mellitus (DM)-induced nephropathy.

**Methods:**

A total of 65 DN patients with proteinuria levels ≥ 2.5 g/24 h and serum creatinine levels < 3 mg/dl were enrolled in this six-month, prospective, randomized, controlled study. The patients were randomized into treatment groups that received either 120 mg of TwHF extract per day for three months, followed by 60 mg per day for three more months, or 160 mg of valsartan daily for six months. The urinary protein and estimated glomerular filtration (eGFR) level were measured at one, three, and six months after the commencement of treatment. The primary measure of treatment efficacy was a reduction in the 24-h urine protein level between baseline and the end of the study, and the secondary measure of treatment efficacy was a reduction in the eGFR value.

**Results:**

At the end of the treatment period, the mean urine protein level in the TwHF group was dramatically decreased (4.99 ± 2.25 g/24 h vs 2.99 ± 1.81 g/24 h, p < 0.01), with decreases at one, three, and six months of 32.9%, 38.8%, and 34.3%, respectively. In contrast, the proteinuria in the valsartan group was not significantly attenuated, and the decreases in urine protein levels at treatment months one, three, and six were 1.05%, 10.1%, and -11.7%, respectively. The mean decrease in eGFR in the valsartan group was greater than that in the TwHF group (26.4% vs. 13.7%, respectively; p =0.067).

**Conclusions:**

TwHF extract can reduce the urine protein level of DN patients and represents a novel, potentially effective, and safe drug for the treatment of DN patients with proteinuria.

**Trial registration:**

ClinicalTrials.gov: NCT00518362

## Background

Diabetes mellitus (DM) is a major public health issue in China. With the rapidly changing lifestyle of the general Chinese population, there is increasing concern that diabetes may become an epidemic [1]. The impact of diabetic nephropathy (DN) in China has been evidenced by our previous studies, in which examinations of renal biopsies revealed that the incidence of DN was 1.68-fold the level over a decade previously [2]. DN has become a common cause of end-stage renal disease (ESRD) in China.

Persistent proteinuria is a hallmark of DN and an independent risk factor for DN progression and DN-related cardiovascular events [3]. Therefore, improvement of proteinuria is as important as control of blood glucose and blood pressure for patients with DN [4]. Several multicenter, prospective, randomized, controlled clinical trials have confirmed that renin-angiotensin system (RAS) blockades with angiotensin II receptor blockers (ARBs) can reduce the urine protein level in patients with DN and may confer additional benefits for renal function [5,6]. However, the efficacy of the currently available ARBs for attenuating proteinuria is insufficient, particularly for DM patients who present with extensive proteinuria and renal dysfunction. Therefore, it is imperative to develop novel strategies to decrease proteinuria to prevent the progression of DN.

*Tripterygium wilfordii* Hook F (TwHF) extract is a traditional Chinese medicine that has been used for many years in glomerulonephritis treatment and organ transplantation [7,8], likely for its immunosuppressive and anti-inflammatory effects [9]. Consistent with this traditional use, Goldbach-Mansky and colleagues have reported that TwHF extract is also effective and safe for the treatment of rheumatoid arthritis [10]. Our recent in vitro and in vivo studies have shown that triptolide (the primary active component of the TwHF extract) protects podocytes from injury [11,12] and ameliorates the albuminuria exhibited by db/db mice, effects that are likely due to its podocyte-protective and anti-inflammatory effects [13].

We conducted a clinical trial to explore the use of TwHF extract in the treatment of DN. This single-center, prospective, randomized, controlled trial was conducted from March 2007 to April 2010 and was registered at ClinicalTrial.gov (identifier: NCT 00518362). The aim of this study was to evaluate the efficacy and safety of TwHF and valsartan for reducing proteinuria in DN patients.

## Methods

### Patient selection

The inclusion criteria were patients 30-65 years of age with a diagnosis of type 2 DM, proteinuria (urine protein ≥ 2.5 g/24 h), and serum creatinine levels of < 3 mg/dl. The diagnosis of DN was confirmed either by the pathological examination of a renal biopsy performed within six months prior to study enrollment or by the presence of clinical manifestations (if a renal biopsy was unavailable). The local ethics committee of Jinling Hospital approved the protocol, and all the enrolled patients provided written informed consent prior to the study. The exclusion criteria included a diagnosis of type 1 DM, nondiabetic kidney disease, liver function impairment (alanine aminotransferase or aspartate aminotransferase levels > twofold the upper limit of normal), a white blood cell (WBC) count < 3.0 × 10^9^/L, severe hypertension (blood pressure > 180/100 mmHg and refractory to treatment), any infections within one month prior to the study, or major cardiovascular and cerebrovascular events (angina pectoris, heart failure, myocardial infarction, cerebral infarction, and cerebral hemorrhage) within the six months prior to the study [5,6,14].

### Treatment plan

During the two-week screening phase [6], patients with hypertension continued to receive the standard antihypertensive therapy. Patients who had been receiving angiotensin-I-converting enzyme inhibitors or angiotensin-II-receptor antagonists were administered alternative medications (diuretics, calcium-channel antagonists, alpha- or beta-blockers, or a combination of these drugs) [5,6,14]. The blood pressure of all the patients was maintained at < 140/90 mmHg [5]. The TwHF extract tablets used in this study were manufactured from the same species (*Tripterygium wilfordii* Hook F) as that used by Goldbach-Mansky et al. [10]. This medicine (10 mg/tablet) was produced by Jiangsu General Pharmaceuticals Co, Ltd, Taizhou, China and was approved by the State Food and Drug Administration (SFDA) of China.

The patients were randomly assigned to two groups. The TwHF group received 120 mg of TwHF per day (one 40-mg tablet three times daily) for three months, followed by 60 mg of TwHF per day (one 20-mg tablet three times daily) for the remaining three months. The valsartan group received 160 mg of valsartan per day (Novartis, two 80-mg capsules once daily) for six months.

During the first three months of TwHF treatment, if liver function impairment or a WBC count < 3.0 × 10^9^/L occurred, the dose was decreased to 60 mg/d for one week. If the patient recovered, then treatment with 60 mg/d TwHF continued. Otherwise, the treatment was discontinued, and the patient was excluded from the study. If a patient developed a severe infection, the TwHF treatment was halted, and the patient was withdrawn from this study. If the infection was resolved within two weeks, then observation continued, but the baseline information would be reevaluated. The dosage of valsartan was decreased to 80 mg/d if either the serum creatinine level increased by 50% and > 2 mg/dl or severe hyperkalemia (K^+^ > 6.0 mmol/L) was observed. The valsartan treatment was discontinued if the above conditions were not controlled by additional treatments. Patients in either group were also excluded from the study if the treatment was discontinued for more than three weeks for any reason.

Throughout the study, the patients received routine care for DM, including measurements of glycosylated hemoglobin (HbA1c) and fasting serum glucose concentrations. The goals of the DM treatment included the maintenance of fasting blood glucose (FBG) levels < 7.0 mmol/L, postprandial blood glucose (PBG) levels < 10.0 mmol/L, and HbA1c levels < 7.0% [15]. Other treatments, such as diuresis to improve edema and albumin infusions, were performed in parallel. However, these treatments were discontinued at least three days prior to the measurement of urinary protein level. No restrictions on dietary salts or proteins were mandated.

### Evaluation of response

Follow-up evaluations were scheduled at one, three, and six months after the randomization (or more frequently, if necessary) to monitor blood pressure, laboratory measurements, and adverse events, as well as to assess end points. Two 24-h urine specimens were obtained over two consecutive days at baseline and one, three, and six months after randomization, and the two-day urine protein measurements were averaged. At baseline and at each follow-up visit, the vital signs and laboratory test results were evaluated, including complete blood count, FBG, blood urea nitrogen (BUN), serum creatinine, estimated glomerular filtration rate (eGFR), serum potassium, serum aspartate aminotransferase, alanine aminotransferase, urinalysis, and 24-h urine protein levels. The 24-h urine protein levels were measured via the trichloroacetic acid method using a photometer [16]. The serum creatinine concentrations were determined by the Jaffe reaction [17]. The Modification of Diet in Renal Disease (MDRD) formula was used to determine the eGFR [18]. The HbA1c levels were measured with high-performance liquid chromatography [19]. All the other laboratory parameters were assessed using conventional laboratory methods. The blood pressure was measured twice while the patient was seated, after at least 15 min of rest, using a standard mercury sphygmomanometer and an appropriately sized cuff. The mean value was calculated for each pair of measurements.

Renal dysfunction was indicated by a serum creatinine level > 1.5 mg/dl, and the progression of renal dysfunction was defined as an increase in serum creatinine of ≥ 25% of the baseline value. The primary measure of treatment efficacy was a reduction in the 24-h urine protein level from baseline until the end of the study period (six months), and the secondary measure of efficacy was a reduction in the eGFR [5,14]. The end points were defined as a doubling of the serum creatinine concentration, the development of ESRD, cardiovascular or cerebrovascular events, or death [6]. The doubling of serum creatinine was defined as a level at the end of the study period that was twice the baseline level and higher than 5 mg/dl. ESRD was defined as serum creatinine > 6 mg/dl, eGFR < 15 ml/min/1.73 m^2^, or the necessity of long-term dialysis [6].

We contacted the patients who did not return for clinical visits to inquire about the reasons for their failure to participate in follow-up, whether they had continued taking the medication, and whether they had reached the study end points.

### Statistical analysis

In our pre-trial, the urine protein level in the TwHF group decreased by 52.2%, whereas the level in the valsartan group decreased by < 10%. A sample size of 46 patients (23 per group) was estimated to be sufficient to provide 80% statistical power to detect a two-sided level of significance of 0.05 at the primary end point. The data are expressed as the mean values ± standard deviation (SD). Student’s t-test, paired t-test, and repeated-measures analysis of variance (ANOVA) were used for between-group comparisons with adjustment for baseline values, as appropriate. Bonferroni correction was performed in t-test analysis. The differences in the urine protein, blood pressure, serum creatinine, serum albumin, and eGFR measurements between baseline and after one, three, and six months of treatment were analyzed by paired t-test, and repeated measures ANOVA was used for the comparisons between TwHF and valsartan group during the follow-ups. The qualitative data are expressed as percentages and were analyzed using the chi-square (*χ*^2^) or Fisher’s exact test, as indicated. The correlations between a decrease in the urine protein level at the end of study period and the blood pressure, urine protein, serum creatinine, and eGFR measurements at baseline were analyzed using the bivariate correlations test. The multiple imputations method and a “pessimistic” analysis were used to assess the impact of missing values. The p-values reported were two-sided, and a p-value < 0.05 was considered statistically significant. All the analyses were performed using SPSS software (version 11.0, SPSS Inc., USA).

## Results

### Patient characteristics

**Figure 1:**
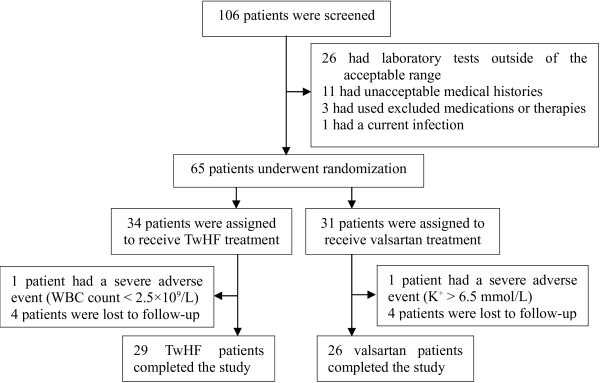
Flowchart of the treatment of DN with TwHF or valsartan.

A total of 106 patients were screened at enrollment, and 41 patients were excluded. The remaining 65 patients were randomized to the TwHF group (34 patients) or the valsartan group (31 patients) and were followed up for at least six months. In the TwHF group, one patient who presented with a WBC count < 2.5 × 10^9^/L was withdrawn from the study, and four other patients were lost to follow-up. In the valsartan group, one patient was withdrawn due to recurrent, severe hyperkalemia (K^+^ > 6.5 mmol/L), and four other patients were lost to follow-up [Figure 1]. The baseline characteristics of the two treatment groups were comparable [Table 1].

**Table 1:** The baseline characteristics of the patients in both groups

	**TwHF group**	**Valsartan group**	**p**
Male: female	20:14	17:14	0.805
Age (years)	51.9 ± 9.8	51.0 ± 8.9	0.784
Duration of DM (months)	126.9 ± 68.1	106.8 ± 57.3	0.270
Duration of DN (months)	33.8 ± 30.6	26.1 ± 28.4	0.277
ACEI or ARB treatment before the screening phase (n,%)	17, 50%	10, 32.3%	0.208
Systolic blood pressure (mmHg)	140.6 ± 15.8	138.3 ± 17.1	0.515
Diastolic blood pressure (mmHg)	81.6 ± 11.9	84.4 ± 11.1	0.233
FBG (mmol/L)	6.04 ± 1.51	6.70 ± 1.62	0.092
Glycosylated hemoglobin (%)	6.26 ± 1.15	6.68 ± 1.26	0.202
Serum albumin (g/L)	33.0 ± 5.66	33.07 ± 4.74	0.988
Total cholesterol (mmol/L)	6.08 ± 1.96	5.92 ± 2.21	0.702
Triglycerides (mmol/L)	1.85 ± 0.83	2.50 ± 2.10	0.111
Serum creatinine (mg/dl)	1.92 ± 0.72	1.67 ± 0.62	0.117
eGFR (ml/min/1.73 m^2^)	43.07 ± 21.65	47.72 ± 20.34	0.377
Serum creatinine > 1.5 mg/dl (%)	70.6%	54.8%	0.210
Urine protein (g/24 h)	4.99 ± 2.25	4.15 ± 1.29	0.097

### Clinical response and adverse events

**Figure 2:**
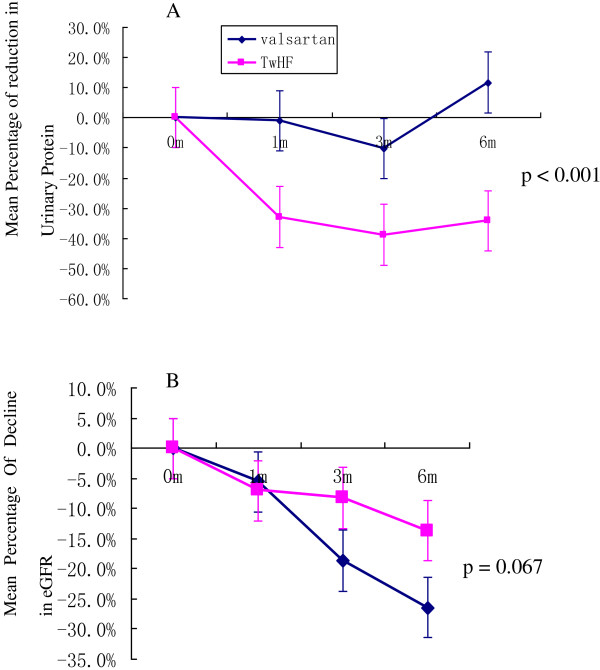
**Changes in the baseline 24-h urine protein and eGFR levels over the six months of TwHF and valsartan treatments. A**: The reductions in the urine protein levels of the available patients, expressed as the mean percentages of urine protein reduction compared with the baseline levels; **B**: The mean reductions in the eGFR values of the available patients, expressed as the mean percentages of eGFR reduction compared with the baseline. Each error bar represents the standard deviation from the mean value. The statistical analysis was performed using repeated-measures ANOVA. (TwHF group vs. valsartan group).

The urine protein levels in the TwHF group decreased dramatically throughout the follow-up period. The reductions from baseline after one, three, and six months of treatment were 32.9%, 38.8%, and 34.3%, respectively. In contrast, the urine protein levels in the valsartan group remained relatively stable, with reductions at months one, three, and six of 1.05%, 10.1%, and -11.7%, respectively (p < 0.001) [Figure 2A]. The changes in the blood pressure, urine protein, serum albumin, serum creatinine, and eGFR during the follow-up period are shown in Table 2. However, there were no significant changes in the serum albumin levels in the TwHF group during the treatment. The mean decreases in eGFR during the six-month treatment period were 26.4% in the valsartan group and 13.7% in the TwHF group (p =0.067) [Figure 2B].

**Table 2:** Changes in the clinical characteristics of the patients in the TwHF group and valsartan group during the follow-up period

	**Baseline**		**Month 1**		**Month 3**		**Month 6**		**P value calculated by repeated-measures ANOVA**		
	**TwHF (n = 34)**	**Valsartan (n = 31)**	**TwHF (n = 33)**	**Valsartan (n = 29)**	**TwHF (n = 33)**	**Valsartan (n = 25)**	**TwHF (n = 29)**	**Valsartan (n = 26)**	**Time**	**Group**	**Time × Group**
Systolic blood pressure (mmHg)	140.6 ± 15.8	138.4 ± 16.6	135.1 ± 14.3	139.4 ± 16.2	137.9 ± 14.6	138.6 ± 19.7	137.4 ± 14.4	141.9 ± 18.4	0.455	0.943	0.408
Diastolic blood pressure (mmHg)	81.6 ± 11.9	85.0 ± 10.8	79.8 ± 10.3	78.7 ± 10.3	79.1 ± 10.0	77.7 ± 8.8	79.5 ± 8.5	76.5 ± 10.5 †	0.03	0.574	0.253
Urine protein (g/24 h)	4.99 ± 2.25	4.15 ± 1.29	3.23 ± 2.57**#	3.92 ± 1.56	2.83 ± 1.57**#	3.59 ± 1.71	2.99 ± 1.81**#	4.40 ± 2.37	<0.001	0.115	0.001
Serum albumin (g/L)	33.0 ± 5.66	33.0 ± 4.69	33.2 ± 5.35 #	37.7 ± 4.25‡	33.9 ± 5.3	37.4 ± 4.64‡	34.8 ± 5.49	36.3 ± 5.47‡	<0.001	0.368	<0.001
eGFR (ml/min/1.73 m^2^)	43.07 ± 21.65	47.72 ± 20.34	38.82 ± 19.93	43.59 ± 17.41	40.23 ± 22.24	39.33 ± 16.79‡	38.71 ± 23.66*	36.22 ± 14.96‡	<0.001	0.682	0.009

* p < 0.05 vs. TwHF baseline; **p < 0.01 vs. TwHF baseline.

† p < 0.05 vs. Valsartan baseline; ‡ p < 0.01 vs. Valsartan baseline.

# p < 0.05 vs. Valsartan group at the same follow-up.

**Figure 3:**
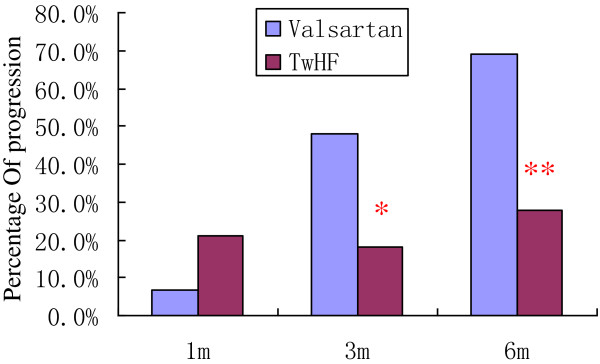
The incidence of renal disease progression during the follow-up period. *p = 0.022 and **p = 0.003 for the TwHF and valsartan groups, respectively.

At months three and six, the proportions of patients exhibiting DN disease progression (as evidenced by serum creatinine levels ≥ 25% higher than baseline) were significantly lower in the TwHF group than in the valsartan group (18.18% vs. 48.00% at month three, respectively, p = 0.022 and 27.59% vs. 69.23% at month six, respectively, p = 0.003) [Figure 3].

A reduction in urine protein ≥ 50% and an increase in serum creatinine < 50% compared with baseline were observed in 31.03% (9 of 29) of the TwHF patients compared with 11.54% (3 of 26) of the valsartan patients (p = 0.076). When the data from the TwHF and valsartan groups were combined, a reduction in urine protein was correlated with the baseline diastolic blood pressure (P = 0.021, R = -0.33) but not with the baseline systolic blood pressure, serum creatinine, urine protein, or eGFR.

A total of ten patients (five in the TwHF group and five in the valsartan group) failed to complete the follow-up evaluations due to loss of contact or severe adverse events. A multiple imputations analysis was performed to assess the impact of the missing values. The recalculated reductions in proteinuria from baseline were 32.2%, 39.1%, and 34.1%, after one, three, and six months of TwHF treatment, respectively, percentages nearly identical to those obtained when the subjects lacking proteinuria data were omitted. The recalculated reductions in eGFR from baseline were 6.75%, 8.71%, and 14.8% in the TwHF group, compared with 6.40%, 18.5%, and 27.2% in the valsartan group after one, three, and six months of treatment, respectively. The results of the pessimistic analysis, which imputed the missing proteinuria values as double the last observed value for the TwHF patients, but equal to the last value for the valsartan patients, indicated reductions in proteinuria of 29.0%, 38.8%, and 26.8% at one, three, and six months, respectively. The proteinuria reductions in the TwHF group were significantly greater than those in the valsartan group. When the missing eGFR values were imputed as half of their last observed value for the TwHF patients, but equal to their last value for the valsartan patients, the reductions in eGFR were 7.08%, 9.61%, and 20.3% in the TwHF group, compared with 5.21%, 15.4%, and 28.3% in the valsartan group at one, three, and six months, respectively (p > 0.05).

The overall incidence of adverse events was 38.4% in the TwHF group, which was nearly identical to the 38.7% incidence observed in the valsartan group. Hyperkalemia necessitated a discontinuation of treatment in one patient (3.22%) in the valsartan group. Treatment was also discontinued for one patient (2.94%) in the TwHF group due to a reduction in the WBC count (< 2.5 × 10^9^/L). Liver function impairment was observed in three patients in the TwHF group. After their TwHF dose was reduced to 60 mg per day, these three patients recovered and finished the trial. Hyperkalemia was observed in eight patients in the TwHF group (with K^+^ > 6.0 mmol/L in three patients) and in ten patients in the valsartan group (with K^+^ > 6.0 mmol/L in two patients). A doubling of the serum creatinine was found in only one patient, who was in the valsartan group. There were no deaths in either group during the follow-up period [Table 3]. The anti-fertility effects of the TwHF extract were not evaluated because the majority of the patients were older than 50, and patients attempting to conceive had been excluded from the study.

**Table 3:** Adverse events during the follow-up period

	**TwHF group (n = 34)**	**Valsartan group (n = 31)**
Any adverse event (n,%)	13 (38.3)	12 (38.7)
Vomiting (n,%)	1 (2.94)	0 (0.00)
Liver dysfunction (n,%)	3 (8.82)	0 (0.00)
Decrease in WBC count (n,%)	1 (2.94)	0 (0.00)
Photosensitive dermatitis (n,%)	0 (0.00)	1 (3.22)
Hyperkalemia (n,%)	8 (23.53)	10 (32.2)
K^+^ > 6.0 (n,%)	3 (8.82)	2 (6.45)
Doubling of serum creatinine (n,%)	0 (0.00)	1 (3.22)

## Discussion

DN is one of the most severe complications of type 2 DM and a major cause of ESRD. The urine protein level not only reflects the degree of renal injury but is also an independent risk factor for the progression of renal disease to ESRD. Increasing numbers of clinical trials have reported a strong association between the baseline level of proteinuria and a decline in eGFR [5,6,20,21]. As a result, urine protein is an important prognostic predictor, and decreased urine protein levels have been considered as a goal of DN treatment.

There were growing evidences indicated that immunologic and inflammatory mechanisms play important roles in the development and progression of DN [22,23]. Inflammatory cytokines such as IL-1, IL-6, IL-18, TNF-α, TGF-β, and MCP-1 have been found to be involved in the pathophysiological processes of DN [22]. High glucose levels, abnormal hemodynamics, immune and inflammatory reactions, and injury of the glomerular basement membrane and podocytes constitute the pathophysiological basis of proteinuria. Indeed, our previous studies have revealed podocyte loss, foot process fusion, slit membrane effacement, the absence of nephrin expression, and the downregulation of WT1 in the glomeruli of DN patients [24,25]. In this regard, apart from strict glycemic control [26], ARBs have been demonstrated to be an effective therapeutic modality for DN [5,6,14,27,28]. The efficacy of ARBs is likely due to their ability to block RAS, to lower blood pressure, to suppress the expression of certain inflammatory cytokines [29], and to protect podocytes [30,31]. Considering the side effects of these drugs, which include hyperkalemia and increased serum creatinine, they are not appropriate for all patients, at least at the currently prescribed dosages. Thus, it is necessary to develop new medications for DN patients with proteinuria.

Previously, we have explored that TwHF extract elicits immune suppressive and anti-inflammatory effects, whereas triptolide potently inhibits NF-κB and the activation of T lymphocytes, as well as promoting apoptosis in activated T lymphocytes [9]. In addition, TwHF can attenuate oxidative stress and inhibit the expression of certain inflammatory cytokines (TNF-α, IL-1β, IL-6, and IFN-γ) [32]. More recently, we further demonstrated that triptolide could protect podocytes against puromycin aminonucleoside-induced injury both in vivo and in vitro, in addition to preventing proteinuria in treated animals [11]. We also found that in cultured murine podocytes, triptolide pretreatment prevented the puromycin-induced disruption of the actin cytoskeleton and microfilament-associated synaptopodin, as well as preventing reductions in nephrin and podocin expression [11,12]. Based on the observed immunosuppressive, anti-inflammatory, and podocyte-protective effects of TwHF extract, we tested the efficacy of TwHF extract in treating diabetic db/db mice and found that albuminuria was markedly attenuated, which was accompanied by an amelioration of glomerular hypertrophy and podocyte injury after triptolide treatment. In addition, we found that the effects of triptolide on renal inflammation and oxidative stress were more profound than those of valsartan [13].

Based on these findings, this prospective, randomized, controlled clinical trial was designed to evaluate the clinical therapeutic efficacy and safety of TwHF extract in patients with DN. We found that the urine protein level could be significantly reduced by TwHF extract. At the end of the trial, the reduction of urine protein in the TwHF group was 34.3%, which was significantly greater than the valsartan group. In addition, the decline in kidney function in the patients receiving TwHF treatment tended to be smaller than that observed in the valsartan-treated patients.

Interestingly, although the urine protein levels were dramatically reduced in the TwHF group, the serum albumin levels did not increase significantly. We speculate that TwHF extract may interfere with the synthesis of albumin; further studies are required to reveal the underlying mechanism.

In this study, an ARB (valsartan) appeared less effective in reducing urine protein levels compared with previous, multicenter trials [5,6]; the urine protein levels in our valsartan group were reduced by only 1.05% and 10.1% at months one and three, respectively, and they tended to increase at month six. This discrepancy might be attributed to the following explanations: 1) seven patients could not tolerate the 160-mg/d valsartan dose due to hyperkalemia and increased serum creatinine; therefore, they switched to a lower dose of 80 mg/d. In contrast, the participants in the RENAAL (losartan 100 mg/d) and IDNT (irbesartan 300 mg/d) trials tolerated higher doses of ARBs. 2) The RENAAL and IDNT trials evaluated the efficacy of losartan or irbesartan rather than valsartan; the therapeutic efficacies of these different ARBs might be distinct. 3) Our inclusion criteria were different from those in the abovementioned trials: our enrollment criterion for proteinuria was ≥ 2.5 g/24 h, and the mean urine protein was 4.15 ± 1.29 (2.51-7.65) g/24 h in the valsartan group, which was significantly higher than that in the RENAAL (urine albumin/creatinine 1,237 ± 1,261 mg/g.cr) and IDNT (median urine protein 2.9 (1.6-5.4) g/24 h) trials. 4) This study investigated the short-term efficacy of TwHF and valsartan treatments with a follow-up period of six months, which was significantly shorter than those of the previous studies.

The target level of blood pressure was < 140/90 mmHg, however, during the follow-ups, 35% patients in TwHF group and 41% patients in valsartan group did not reach the target level. This discrepancy might be mostly attributed to the high percentage (63%) of the patients suffered from chronic renal insufficiency. Although the blood pressure did not touch the target, the systolic blood pressure and diastolic blood pressure levels between two groups either at the baseline or during the follow-ups did not show significant difference. So it is not a major factor that impacts the efficacy judgement.

Regarding eGFR, Nelson et al. have reported that diabetic Pima Indians with macroalbuminuria demonstrated eGFR declines of only 1 ml/min/1.73 m^2^ per month over a four-year period [33], in contrast to the larger change in eGFR over the six-month study period in this trial. This discrepancy might be attributed to the proteinuria and serum creatinine levels of the patients of this study, which were both significantly higher than those in the Nelson et al. study. Moreover, most of the patients enrolled in our trial were in the late stages of DN, whereas in the study by Nelson et al., all of the patients presented with normal renal function.

Because ten patients were withdrawn from the present study, we conducted several analyses to specifically assess the impact of the missing data, including a multiple imputations analysis and a pessimistic analysis of the missing values. The qualitative and quantitative proteinuria results of these two analyses were similar to those of the primary analyses (with the missing values omitted). We also recalculated the eGFR values using these two methods. Based on these exploratory analyses, we concluded that the missing values due to patient withdrawal produced no significant impact on our conclusions concerning the primary efficacy of the TwHF extract treatment evaluated in this clinical trial.

In previous clinical trials of TwHF, the major adverse events included nausea, vomiting, loss of appetite, decreased WBC count, and liver function impairment [7,8]. Several Studies have reported the potential mechanisms of hepatoxicity and leucopenia induced by TwHF [34-36]. In this trial, we found that the TwHF extract dose of 120 mg/d was well tolerated by the majority of the patients. Although some adverse events occurred in the TwHF group, their incidence was comparable to that observed in the valsartan group. Previous clinical trials have also reported detrimental effects of TwHF extract on the genital system [8], including menstrual disorders in female patients. However, these effects were not observed in this trial, which was largely due to the older age (> 50 years) of the majority of the female patients.

This trial was limited by the following factors: 1) it was a single-center trial with a small sample size and a relatively short follow-up period, 2) the treatment assignment was not blinded, 3) the rate of subject withdrawal during the follow-up period was > 10%, and 4) a reduction in the 24-h urine protein level was used as a surrogate end point. Thus, a multicenter, blinded, long-term study is needed to confirm the renoprotective effects of TwHF extract in patients with DN.

## Conclusions

In conclusion, TwHF extract can reduce the urine protein levels of patients with DN and thus represents a novel, potentially effective, and safe renoprotective drug for the treatment of DN. TwHF extract may play an important role in multidrug regimens for the treatment of DN.

## Abbreviations

DN: Diabetic nephropathy; ARBs: Angiotensin II receptor blockers; TwHF: *Tripterygium wilfordii* Hook F; DM: Diabetes mellitus; eGFR: Estimated glomerular filtration; ESRD: End-stage renal disease; RAS: Renin-angiotensin system; WBC: White blood cell; HbA1c: Glycosylated hemoglobin; FBG: Fasting blood glucose; PBG: Postprandial blood glucose; BUN: Blood urea nitrogen; MDRD: Modification of diet in renal disease; SD: Standard deviation; ANOVA: Analysis of variance; TW: Tripterygium wilfordii.

## Competing interests

The authors declare that they have no competing interests.

## Authors’ contributions

YC Ge conceived the study, participated in its design and coordination, and drafted the manuscript. HL Xie and SJ Li interpreted the data and helped to draft the manuscript. B Jin, JH Hou, and HT Zhang participated in the design of the study and performed the statistical analyses. MJ Shi performed the laboratory experiments. ZH Liu supervised the study and participated in its design. All the authors read and approved the final manuscript.
